# Efficient Hydrogen Evolution Reaction with Bulk and Nanostructured Mitrofanovite Pt_3_Te_4_

**DOI:** 10.3390/nano12030558

**Published:** 2022-02-06

**Authors:** Gianluca D’Olimpio, Lixue Zhang, Chia-Nung Kuo, Daniel Farias, Luca Ottaviano, Chin Shan Lue, Jun Fujii, Ivana Vobornik, Amit Agarwal, Piero Torelli, Danil W. Boukhvalov, Antonio Politano

**Affiliations:** 1 Department of Physical and Chemical Sciences, University of L’Aquila, Via Vetoio, 67100 L’Aquila, Italy; gianluca.dolimpio@univaq.it (G.D.); luca.ottaviano@aquila.infn.it (L.O.); 2 College of Chemistry and Chemical Engineering, Qingdao University, Qingdao 266071, China; zhanglx@qdu.edu.cn; 3 Department of Physics, National Cheng Kung University, 1 Ta-Hsueh Road, Tainan 70101, Taiwan; kuochianung@gmail.com (C.-N.K.); cslue@ncku.edu.tw (C.S.L.); 4 Taiwan Consortium of Emergent Crystalline Materials, Ministry of Science and Technology, Taipei 10601, Taiwan; 5 Departamento de Física de la Materia Condensada, Universidad Autónoma de Madrid, 28049 Madrid, Spain; daniel.farias@uam.es; 6 Instituto “Nicolás Cabrera”, Campus de Cantoblanco, 28049 Madrid, Spain; 7 Condensed Matter Physics Center (IFIMAC), Universidad Autónoma de Madrid, 28049 Madrid, Spain; 8 CNR-SPIN, Uos L’Aquila, Via Vetoio 10, 67100 L’Aquila, Italy; 9 CNR-IOM, TASC Laboratory, Area Science Park-Basovizza, 34149 Trieste, Italy; jun.fujii@elettra.eu (J.F.); ivana.vobornik@elettra.eu (I.V.); piero.torelli@elettra.eu (P.T.); 10 Department of Physics, Indian Institute of Technology Kanpur, Kanpur 208016, India; amitag@iitk.ac.in; 11 College of Science, Institute of Materials Physics and Chemistry, Nanjing Forestry University, Nanjing 210037, China; danil@njfu.edu.cn; 12 Theoretical Physics and Applied Mathematics Department, Ural Federal University, Mira Street 19, 620002 Ekaterinburg, Russia; 13 CNR-IMM, Istituto per la Microelettronica e Microsistemi, VIII Strada 5, 95121 Catania, Italy; 14 INSTM, University of L’Aquila Unit, 67100 L’Aquila, Italy

**Keywords:** metal chalcogenides, hydrogen evolution reaction, electrocatalysis

## Abstract

Here, we discuss the key features of electrocatalysis with mitrofanovite (Pt_3_Te_4_), a recently discovered mineral with superb performances in hydrogen evolution reaction. Mitrofanovite is a layered topological metal with spin-polarized topological surface states with potential applications for spintronics. However, mitrofanovite is also an exceptional platform for electrocatalysis, with costs of the electrodes suppressed by 47% owing to the partial replacement of Pt with Te. Remarkably, the Tafel slope in nanostructured mitrofanovite is just 33 mV/dec, while reduced mitrofanovite has the same Tafel slope (36 mV/dec) as state-of-the-art electrodes of pure Pt. Mitrofanovite also affords surface stability and robustness to CO poisoning. Accordingly, these findings pave the way for the advent of mitrofanovite for large-scale hydrogen production.

## 1. Introduction

In recent years, renewable sources have played a pivotal role in the context of energy production, constituting a valid alternative to nonrenewable energy sources now close to exhaustion [1,2,3]. On the other hand, renewable energy sources are inexhaustible and have a low environmental impact. Accordingly, the capability to meet world energy demand in a sustainable way, minimizing both dependence on the unstable fossil fuel market and associated emissions, is one of the main challenges of the 21st century.

In this context, hydrogen could play a key role in the future of energy devices and their related implications for economy [4,5,6,7]. Hydrogen is the most abundant light element of the universe. Its combustion reaction has a high calorific value, with the production of only water, avoiding the formation of greenhouse gases. It should not be considered as an energy source, but rather as an energy carrier, that is, a compound capable of conveying energy from one form to another.

Energy produced from renewable sources through water splitting can be stored through the formation of chemical bonds that are formed by the splitting of the H_2_O molecule [8,9]. Nowadays, 3% of the world’s hydrogen production concerns the electrolysis of water. Produced hydrogen can be stored, transported, and for energy purposes it can be used in fuel cells with the purpose of producing electricity again.

The water splitting reaction is endothermic and therefore requires energy that can be supplied by the flow of an electric current through an electrochemical cell.

2H_2_O (aq) + Energy → 2H_2_ (g) + O_2_ (g) (1)

Water splitting is a process consisting of two reactions, the hydrogen evolution reaction (HER) [10,11] and the oxygen evolution reaction (OER) in which two catalysts are required, one for the oxidation of water into O_2_, and another for the reduction of protons to H_2_.

In the cathodic part of Equation (1) HER takes place:

2H_2_O + 2e^−^ → H_2_ + 2OH^−^ (2)

2H^+^ + 2e^−^ → H_2_ (3)

In a basic and acidic electrolyte, respectively.

HER begins with the Volmer step, which involves the bond of hydrogen to the electrode at an adsorption site M:

M + H_2_O + e^−^ → MH_ads_ + OH^−^ (4)

M + H^+^ + e^−^ → MH_ads_ (5)

And it is completed with a desorption step that takes place through the Tafel reaction:

MH_ads_ → 2M + H_2_ (6)

Or via Heyrovsky’s reaction:

H_2_O + MH_ads_ + e^−^ → M + H_2_ + OH^−^ (7)

MH_ads_ + H^+^ + e^−^ → H_2_ + M (8)

Thus, HER is a reduction reaction (hydrogen goes from an oxidation state +1 to 0) so it will take place at the cathode.

Definitely, green hydrogen production has enormous potential in providing a cycle of use of sustainable energy and opening a new paradigm for different industrial sectors that today mainly depend on fossil fuels and for which decarbonization is challenging. Electrolytic production of hydrogen faces technological challenges to develop scalable methods with inferior energy consumption. The key point is to reduce the cost of raw materials used as electrocatalysts currently based on pure platinum (Pt). Thus, the production of high purity hydrogen requires low-cost electrocatalysts, with relatively low loading of noble metals in electrodes.

Despite the excellent performances of Pt in HER, the high cost (>30 US $/g) and the restricted obtainability of Pt make unavoidable the quest of economic and earth-abundant potential alternatives [12,13,14,15]. One possible solution is to reduce Pt content by using Pt-based alloys [14,16].

Considering its crucial technological relevance, the identification of efficient electrocatalysts for HER with high activity, cost-effectiveness, and long-term stability represents one of the most important open challenges in electrochemistry.

In particular, mitrofanovite is a recently discovered mineral [17], which was demonstrated to be a topological metal with spin-polarized surface states, with subsequent capabilities for applications in spintronics [18]. Nevertheless, recent findings from different groups highlight the huge potential of mitrofanovite for hydrogen production [19,20]. In this Feature Article, the main features of electrocatalysis with mitrofanovite are discussed, with a particular focus on the effects of dimensionality on the catalytic performances. Moreover, we provide a comparison with parental compounds to highlight the capabilities of mitrofanovite.

## 2. Materials and Methods

Single crystals of Pt_3_Te_4_ were grown from the self-flux method. The mixtures of high purity Pt foil and Te ingots with a molar ratio of 51:49 were placed in an alumina crucible and sealed into a quartz ampoule. The quartz ampule was heated to 1080 °C for 24 h, then slowly cooled at a rate of 1 °C/h up to 970 °C. The excess flux was separated by centrifugation.

Micro-Raman spectra were acquired with a He–Ne laser source (λ = 632.8 nm) with a LABRAM spectrometer equipped with an optical microscope with a 100x MPLAN with numerical aperture of 0.9. The system operates in a back-scattering configuration and all the measure were carried out at room temperature.

X-ray photoelectron spectroscopy (XPS) measurements were performed at the Advanced Photoelectric Experiments-HE (APE-HE) beamline of the Elettra synchrotron in Trieste, Italy. XPS spectra were acquired at room temperature and in normal emission with an Omicron EA125 hemispherical analyser.

Atomic force microscopy (AFM) images were acquired with a Digital D5000, Veeco system operating in Tapping-mode.

Theoretical methods to calculate HER and OER are reported in Section S1 of the Supporting Information.

High-resolution electron energy loss spectroscopy (HREELS) experiments were carried out with an Ibach-type spectrometer with an energy resolution of 0.5 eV. The primary electron beam energy was 4 eV. All spectra were taken at room temperature and in specular geometry, with an incident angle of 55° with respect to the sample normal.

The synchrotron X-ray diffraction (SXRD) patterns were collected from 100 to 480 K with the MYTHEN detector with 15 keV beam at beam line 09A, Taiwan Photon Source, National Synchrotron Radiation Research Center (NSRRC) in Hsinchu, Taiwan. The single crystal was pulverized and packed in a 0.1 mm borosilicate capillary to minimize the absorption effect. The capillary was kept spinning during data collection for powder averaging.

Electrochemical tests were carried out on a Bio-Logic VSP-300 electrochemical workstation with a typical three-electrode system, in which a bulk Pt_3_Te_4_ plate, a Pt wire and a saturated Ag/AgCl were used as the working electrode, the counter electrode, and the reference electrode, respectively. The inherent electrochemical behaviour of Pt_3_Te_4_ was in 0.05 M phosphate buffered saline electrolyte (pH 7.0) at a scan rate of 50 mV s^−1^. For HER tests, the polarization curves were obtained using LSV in 0.5 M H_2_SO_4_ at a scan rate of 2 mV s^−1^. The chronopotentiometric test was performed in 0.5 M H_2_SO_4_ at a potential of −0.053 V (vs RHE).

## 3. Results and Discussion

**Figure 1:**
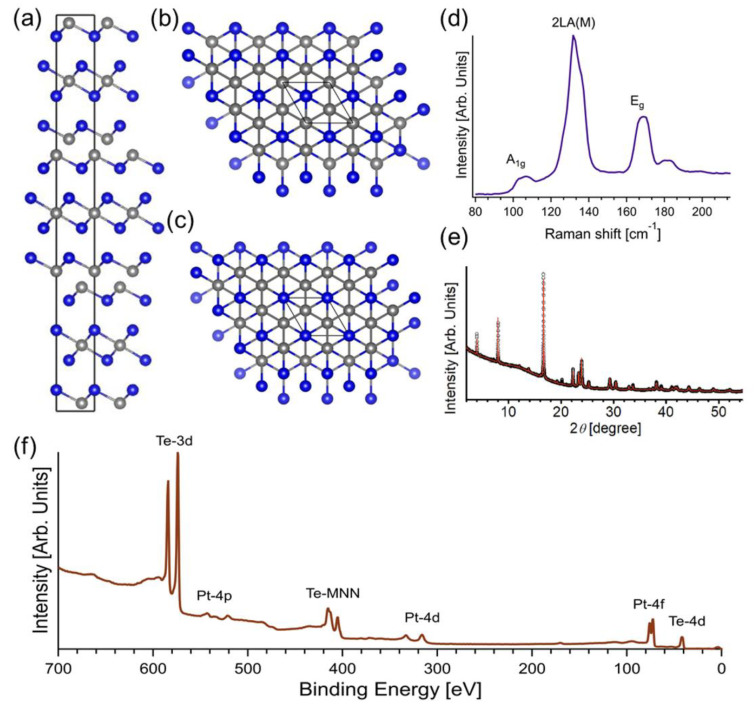
(**a**) Side view of Pt_3_Te_4_ crystal structure, with alternate PtTe_2_ and Pt_2_Te_2_ layers stacked along the vertical direction. Panels (**b**,**c**) show the top view of Pt_3_Te_4_ with Pt_2_Te_2_ and PtTe_2_ terminations, respectively. (**d**) Raman spectra acquired using a laser with λ = 632.8 nm. (**e**) SXRD pattern acquired at T = 300 K, together with the simulated SXRD pattern for Pt_3_Te_4_. (**f**) Survey spectrum of as-cleaved Pt_3_Te_4_ acquired with photons of 730 eV.

The crystal structure of Pt_3_Te_4_ was formed by two different alternate Pt_2_Te_2_ and PtTe_2_ sublayers, bonded along the vertical direction by a combination of electrostatic and van der Waals non-covalent forces (Figure 1a–c). In the PtTe_2_ (Pt_2_Te_2_) sublayer, one (two) Pt atomic layers is (are) sandwiched between two Te atomic layers. Specifically, mitrofanovite belongs to the R-3m (No.166) space group with calculated lattice parameters equal to a = 3.99 Å and c = 35.40 Å. The single crystal of Pt_3_Te_4_ displays three Raman active modes at ~107, 132, and 169 cm^−1^, assigned to A_1g_, 2LA(M) and E_g_ phonons, respectively (Figure 1d). To identify the phase, we carried out SXRD experiments, using a synchrotron-based facility to reduce the broadening of peaks compared to in-house XRD systems. All diffraction peaks in Figure 1e match with the Pt_3_Te_4_ structure (ICSD # 41372). Our single crystals did not display any minimal trace of contamination, as demonstrated by the XPS survey acquired with synchrotron radiation to enhance surface sensitivity and energy resolution (Figure 1f).

**Figure 2:**
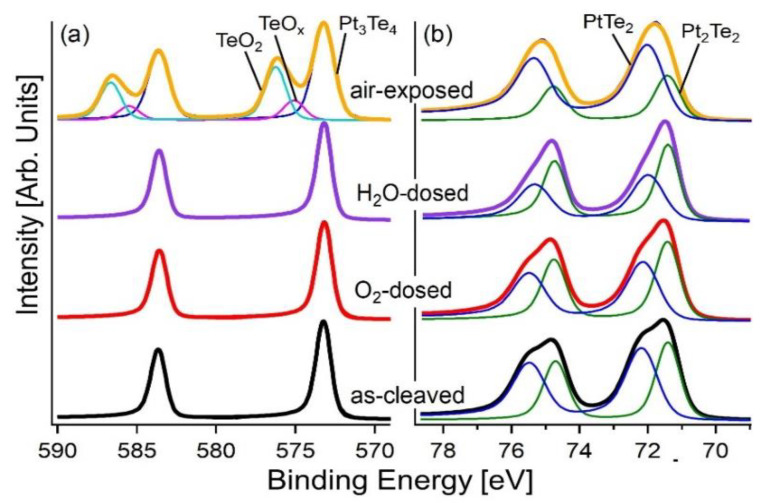
(**a**) Te-3d and (**b**) Pt-4f core level spectra of Pt_3_Te_4_ single crystal. The crystal was exfoliated in UHV to prevent oxidation and subsequently exposed to 10^4^ L (1 L = 10^−6^ Torr·s) of oxygen (red spectra) and water (violet spectra). The yellow spectra represent the air exposed sample. The photon energy is 900 eV, all the spectra are normalized to the maximum.

To study the surface chemical reactivity of single crystal Pt_3_Te_4_, we carried out synchrotron-based XPS experiments, whose superior energy resolution compared to standard XPS could unravel core-level shifts inaccessible with laboratory X-ray sources. Specifically, we studied the evolution of the surface chemical reactivity in oxygen and humid environment and finally in ambient atmosphere (Figure 2). The Pt-4f core level spectra was split into two components related to Pt-4f_7/2_ and Pt-4f_5/2_ shifted by 3.3 eV. The Pt-4f_7/2_ component was characterized by two components located at binding energies (BEs) of 71.5 and 72.3 eV, respectively (Figure 2b). Actually, the presence of these two well-distinct spectral components was due to the PtTe_2_ and Pt_2_Te_2_ sub-units [18]. The Te-3d core level was characterized by one doublet shifted by 10.4 eV, with the binding energy of Te-3d_5/2_ peak of 573.1 eV (Figure 2a).

After the modification of the surface towards 10^4^ L of oxygen and water, no changes in the core levels of Pt and Te were observed. Only after air exposure did the Te-3d core level show the emergence of two new features with BEs of 575.7 and 573.8 eV for the J = 5/2 component, corresponding to TeO_2_ and Te(0), respectively (Figure 2a). Moreover, the Te termination evolved into a nanometric oxide layer, whose thickness was estimated by quantitative XPS. Conversely, in the case of the Pt_3_Te_4_-nanocrystal, tellurium–oxide was always present [20].

**Figure 3:**
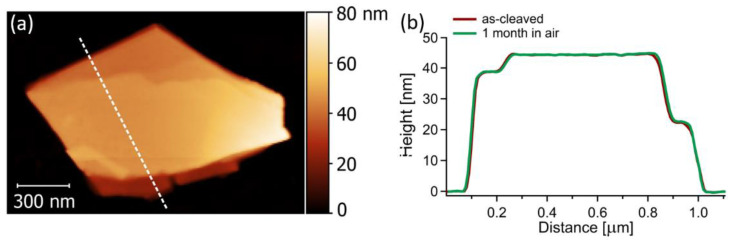
(**a**) AFM image of a 50 nm thick flake of Pt_3_Te_4_. (**b**) height profile taken along the dotted white line in (**a**).

Furthermore, the environmental stability of Pt_3_Te_4_ was studied through the analysis of the time evolution of AFM images (Figure 3a,b). In details, in Figure 3b the height profile along a specific direction remained constant during the air exposure, demonstrating that the morphology of the surface was unchanged. The relative stability of the surface of mitrofanovite made it a suitable candidate as an electrocatalyst.

**Figure 4:**
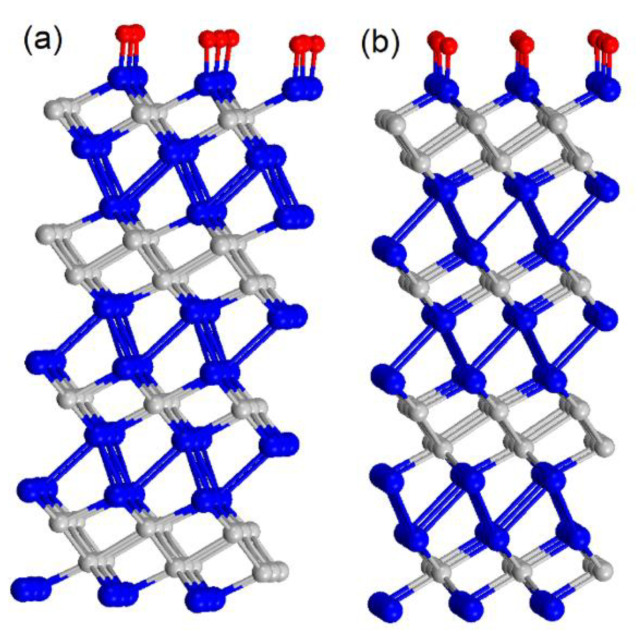
Optimized atomic structure of oxidized PtTe_2_ (**a**) and Pt_2_Te_2_ (**b**) sides of Pt_3_Te_4_.

Theoretical modelling indicates that physisorption and further decomposition of molecular oxygen were exothermic processes for both PtTe_2_ and Pt_2_Te_2_ surfaces (Table 1). Step-by-step modelling of the oxidation process demonstrated that only Te atoms were oxidized on the surface layers of Pt_3_Te_4_ (Figure 4), in perfect agreement with the experimental results (Figure 2). Note that Pt centered on the surfaces of Pt_3_Te_4_ remained available for interaction with hydrogen even after oxidation.

**Figure 5:**
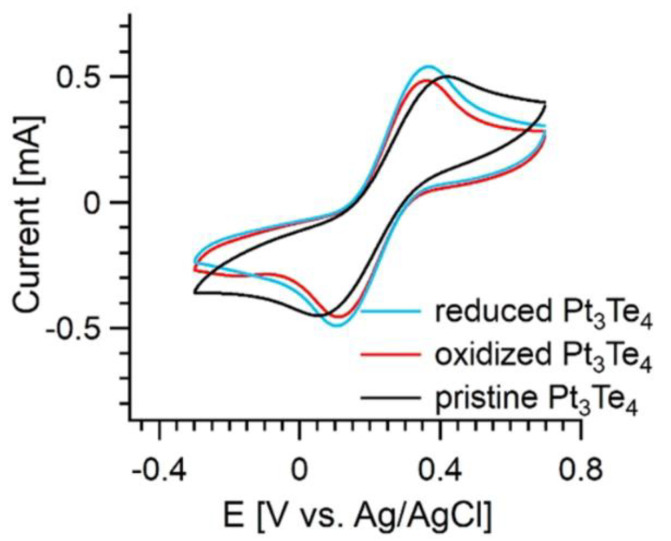
Cyclic voltammograms of the pristine and the electrochemically treated Pt_3_Te_4_ in 0.1 M KCl solution containing 5 mM [Fe(CN)_6_]^3−/4−^ at a scan rate of 50 mV s^−1^. Reproduced with permission from Ref. [19].

In Figure 5, we report the cyclic voltammetry tests by directly using Pt_3_Te_4_ bulk plate as the working electrode. Unlike PtTe_2_, under neutral conditions, the evolution of hydrogen in Pt_3_Te_4_ becomes prominent for potential values below −1.35 V (vs. Ag/AgCl), while for PtTe_2_, it is not prominent up to potential values below −1.8 V (vs. Ag/AgCl) [21,22], this reveals a very promising catalytic activity for Pt_3_Te_4_. Furthermore, in Figure 5, we show the cyclic voltammetry tests on the modified surface of Pt_3_Te_4_ to deeply study the heterogeneous electron transfer. Considering that Pt_3_Te_4_ undergoes redox behavior under different applied potentials, both the pristine Pt_3_Te_4_ and the same system modified by different electrochemical treatments were investigated. Herein, commonly used [Fe(CN)_6_]^3−/4−^ was selected as the redox probe. Definitely, both the oxidizing treatment (1.3 V (vs. Ag/AgCl) for 5 min) and reducing treatment (−1.5 V (vs. Ag/AgCl) for 5 min) had a positive effect and the cyclic voltammogram curves of [Fe(CN)_6_]^3−/4−^ on the pristine, oxidized, and reduced Pt_3_Te_4_ were similar. Remarkably, the electrochemical redox behavior of [Fe(CN)_6_]^3−/4−^ on the oxidized and reduced Pt_3_Te_4_ were even more reversible.

To compare the effectiveness of mitrofanovite for HER, we adopt as figures of merit (i) the Tafel slope (to assess kinetics at the electrode/electrolyte interface) and (ii) η10, i.e., the overpotential required to attain a cathodic current density of 10 mA cm^−2^ per geometric area. The Tafel slope estimates the potential increase necessary to increase the current density by one order of magnitude. In the case of an insufficient coverage of hydrogen atoms H_ads_, the Volmer reaction would be the rate limiting step for the HER, resulting in a theoretical Tafel slope around 120 mV/dec. Conversely, for the highest values of H_ads_, the HER-kinetic is dominated by the Heyrovsky or Tafel reaction, resulting in Tafel slopes around 30–40 mV/dec.

**Figure 6:**
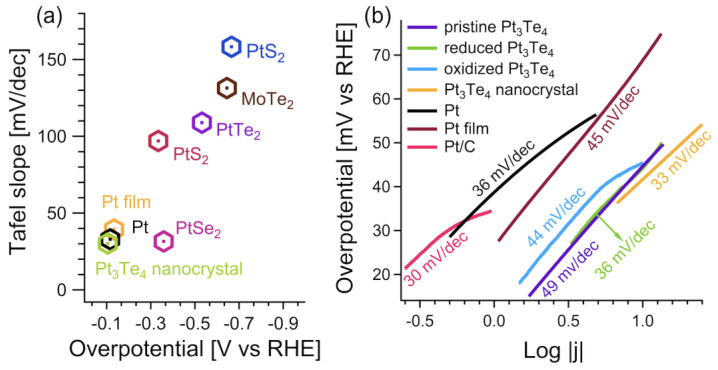
(**a**) Tafel slope vs overpotential for Pt_3_Te_4_ [19,20], MoTe_2_ [20], Pt film [20], platinum dichalcogenides [22,23,24,25], platinum [27], and Pt/C [19]. (**b**) Comparison of Tafel plots for different Pt_3_Te_4_ crystals. Purple, green, light blue, and yellow lines represent pristine Pt_3_Te_4_, reduced Pt_3_Te_4_, oxidized Pt_3_Te_4_, and Pt_3_Te_4_ nanocrystals, respectively. For the sake of comparison, we also report Pt (black), Pt film (violet) and Pt/C (magenta). The figure is built using data from Refs. [19,20], and the spectra was renormalized to simplify the visualization.

In Figure 6, we compare the performances of mitrofanovite for HER with other Pt-based chalcogenides, such as PtS_2_, PtSe_2_, and PtTe_2_. The pristine Pt_3_Te_4_ has a Tafel slope of ~49 mV/dec, while this value is modified in ~36 and 44 mV/dec for the reduced and oxidized surface of Pt_3_Te_4_, respectively. Note that the value of the Tafel slope in the reduced Pt_3_Te_4_ is comparable with the pristine Pt. Moreover, for all Pt_3_Te_4_ systems, including the oxidized surface, the Tafel slope is inferior to Pt-based dichalcogenides PtS_2_, PtSe_2_, and PtTe_2_ (Figure 6a) [22,23,24,25,26] with a comparable value to those of Pt films and Pt/C [27]. Thus, the electrochemical treatment of the Pt_3_Te_4_ surface improves its performance as an electrocatalyst, and this can be correlated to an increase in the heterogeneous electron transfer capability. Additionally, from the inspection of Figure 6b it is evident that the Tafel slope of Pt_3_Te_4_ decreases with the dimensionality. In fact, in the case of Pt_3_Te_4_ nanocrystals, the reported Tafel slope was 33 mV/dec and the reported current density was as high as 7000 mA/cm^2^, which should be attributed to the formation of numerous edges and defects in the growth process and the consequent near-zero Gibbs free-energy change of hydrogen adsorption [20].

Moreover, unlike other transition-metal dichalcogenides (TMDs) with high performance in HER and low current densities of 10–100 mA/cm^2^, Pt_3_Te_4_ exhibits an overall current density, which exceeded 1000 mA/cm^2^, i.e., a large value enabling in principle large-scale hydrogen production [28,29,30,31].

**Figure 7:**
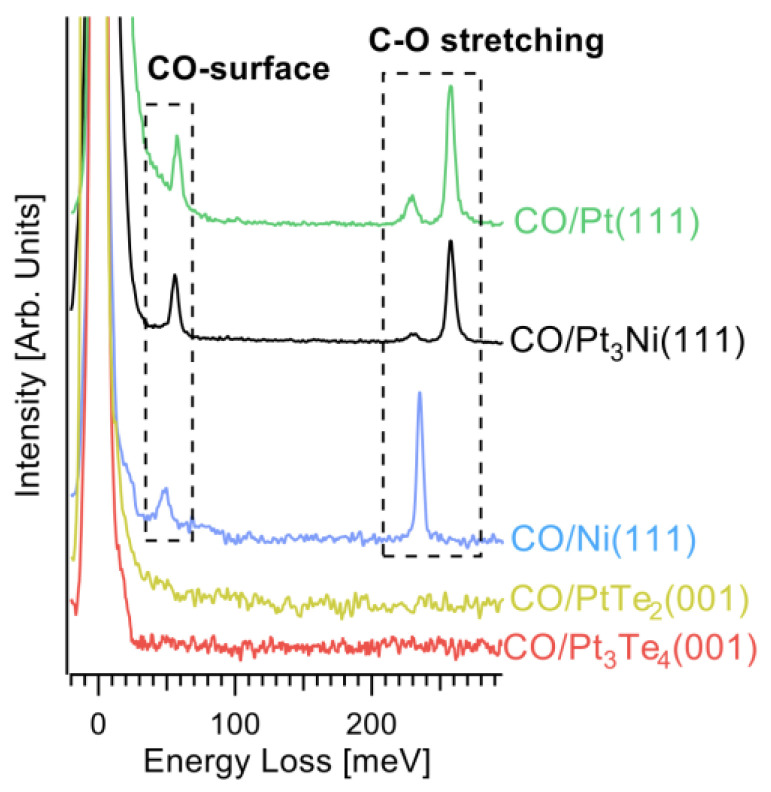
Vibrational spectra after having saturated with CO the surfaces of Pt_3_Te_4_(001), PtTe_2_(001), Ni(111), Pt_3_Ni(111) and Pt(111). The saturation has been reached at only 5, 10, and 8 L for Ni(111), Pt_3_Ni(111), and Pt(111). On the other hand, no CO-derived features are achieved even after exposure to 1010 L on Pt_3_Te_4_(001) and PtTe_2_(001). Specifically, the CO-derived features are the vibration of the whole CO molecule against the substrate at 50 meV [32,33] and the intramolecular C-O stretching [32], whose energy depends on the adsorption site: 230 meV for three-fold site, selectively occupied on Ni, while it is a minority on Pt_3_Ni(111) and Pt(111), and 250 meV for the on-top adsorption site, a majority on both Pt_3_Ni(111) and Pt(111).

Notably, vibrational data (Figure 7) demonstrated that Pt_3_Te_4_ showed outstanding tolerance to CO (contrary to pure Pt, which suffers CO poisoning) and stability in a water environment, with subsequent high suitability for HER in both acidic and alkaline conditions. Physisorption of CO on Te vacancies was even more energetically unfavorable than on defects-free surfaces (+3.74 and +6.76 kJ/mol for the PtTe_2_ and Pt_2_Te_2_ terminations of the Pt_3_Te_4_ surface, respectively).

To validate robustness to CO poisoning, we carried out high-resolution electron energy loss spectroscopy experiments, which is especially sensitive to CO adsorption (see our review for CO adsorption on catalytic surfaces [32] for more details), due to the high oscillating dipole. We carried out the experiment in specular conditions in order to maximize the sensitivity to dipole oscillations. Explicitly, we dosed CO onto (i) Pt_3_Te_4_(001); (ii) PtTe_2_(001); (iii) Ni(111); (iv) Pt_3_Ni(111); and (v) Pt(111) surfaces. We obtained that, while Ni(111), Pt_3_Ni(111) and Pt(111) are poisoned by CO, as evidenced by the observation of C-O stretching and the CO-substrate vibrations, PtTe_2_ and Pt_3_Te_4_ are totally inert toward CO, even after prolonged CO exposure up to 10^10^ L (Figure 7).

**Figure 8:**
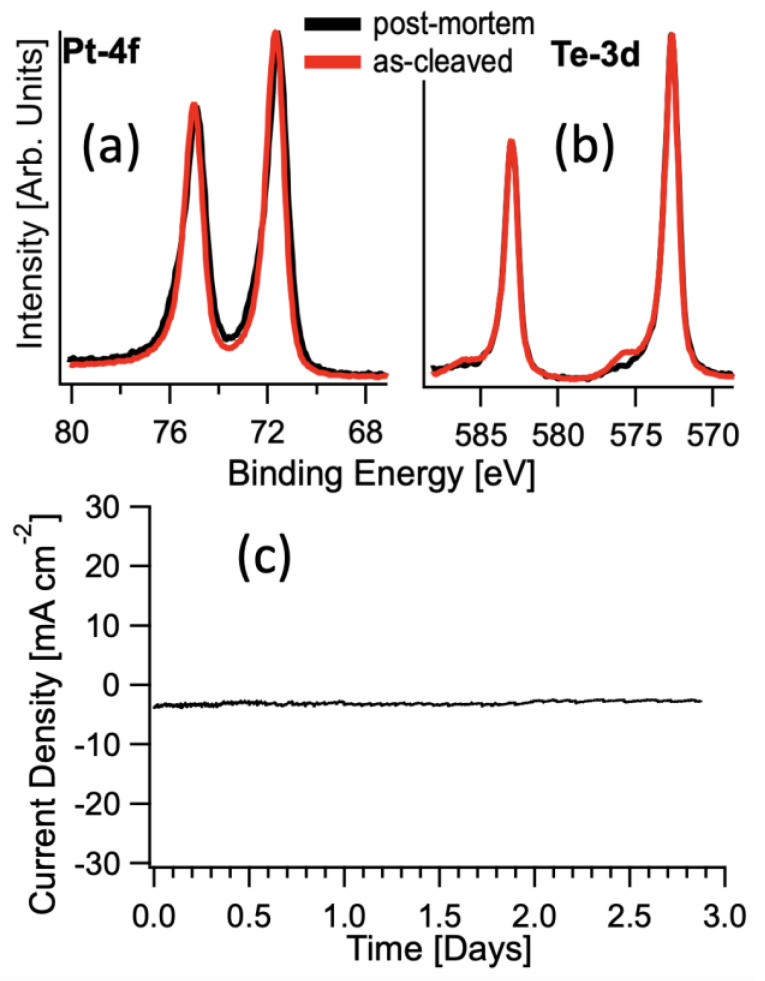
(**a**) Te-3d and (**b**) Pt-4f core level spectra of Pt_3_Te_4_ single crystal for as-prepared and postmortem electrodes. (**c**) Chronopotentiometric curve for bulk Pt_3_Te_4_ in 0.5 M H_2_SO_4_ at a potential of −0.053 V vs. RHE.

To obtain more information on the stability of Pt_3_Te_4_ electrodes in the electrochemical environment, Pt-4f (Figure 8a) and Te-3d (Figure 8b) core levels were measured on both as-prepared and postmortem electrodes. From the inspection of Figure 8, it is quite evident that Pt_3_Te_4_ electrodes were not affected by degradation after electrochemical treatment, with just negligible changes. The only change is in the Te core level where we observe a lower component of tellurium oxide after the reaction. On the other hand, the electrochemical stability is a crucial aspect in electrocatalysis. The chronopotentiometric curve (Figure 8c) in 0.5 M H_2_SO_4_ at a potential of −0.053 V vs RHE, showing negligible attenuation in a timescale extended up to three days further validates the outstanding chemical and electrocatalytic stability of Pt_3_Te_4_.

## 4. Conclusions

In this paper, we discussed the key features ruling electrocatalysis with mitrofanovite (Pt_3_Te_4_), a recently discovered mineral with superb performances in HER.

Mitrofanovite has a surface stable even upon exposure to ambient atmosphere, with just a sub-nanometric oxide skin.

Mitrofanovite represents an exceptional platform for electrocatalysis, with costs of the electrodes suppressed by 47% owing to the partial replacement of Pt with Te. Remarkably, the Tafel slope in nanostructured mitrofanovite is just 33 mV/dec, while reduced mitrofanovite has the same Tafel slope (36 mV/dec) than state-of-the-art electrodes of pure Pt. Contrary to Pt, mitrofanovite is not affected by CO poisoning.

Accordingly, these results pave the way for the advent of mitrofanovite for large-scale hydrogen production.

## Figures and Tables

**Table 1:** Differential enthalpy ΔH_ads_ and differential Gibbs free energy ΔG_ads_ for physical adsorption and differential enthalpy of decomposition ΔH_dec_ for molecular oxygen on PtTe_2_- and Pt_2_Te_2_- terminated Pt_3_Te_4_ surfaces.

Adsorbent	SurfaceTermination	Site	ΔH_ads_ [kJ/mol]	ΔG_ads_ [kJ/mol]	ΔH_dec_ [kJ/mol]
O_2_	PtTe_2_	on-top	−42.7	−31.2	−51.8
		Te vacancy	−33.3	−22.0	−69.4
	Pt_2_Te_2_	on-top	−40.9	−29.6	−98.1
		Te vacancy	−35.0	−23.7	−163.0

## Data Availability

Data are available pending reasonable requests to the corresponding authors.

## Supplementary Materials

The following supporting information can be downloaded at: https://www.mdpi.com/article/10.3390/nano12030558/s1, Section S1: Theoretical Methods. Refs. [34,35,36,37,38,39]
