# Supplementary material for: Efficient Hydrogen Evolution Reaction with Bulk and Nanostructured Mitrofanovite Pt3Te4
Source: Nanomaterials (Basel). 2022 Feb 6;12(3):558. doi: 10.3390/nano12030558 (PMC8839345; doi:10.3390/nano12030558)
Supplement: Supplementary file 1 [file nanomaterials-12-00558-s001.zip › nanomaterials-1551909-supplementary.pdf]

Supplementary Materials

Gianluca D'Olimpio[1], Lixue Zhang[2], Chia-Nung Kuo[3,4], Daniel Farias[5,6,7], Luca Ottaviano[1,8], Chin Shan Lue[3,4], Jun Fujii[9], Ivana Vobornik[9], Amit Agarwal[10], Piero Torelli[9], Danil W. Boukhvalov[11,12], Antonio Politano[1,13,14,*]

[1] Department of Physical and Chemical Sciences, University of L'Aquila, via Vetoio, 67100 L'Aquila (AQ), Italy
[2] College of Chemistry and Chemical Engineering, Qingdao University, Qingdao 266071, Shandong, China
[3] Department of Physics, National Cheng Kung University, 1 Ta-Hsueh Road, 70101 Tainan, Taiwan
[4] Taiwan Consortium of Emergent Crystalline Materials, Ministry of Science and Technology, Taipei 10601, Taiwan
[5] Departamento de Física de la Materia Condensada, Universidad Autónoma de Madrid, 28049 Madrid, Spain
[6] Instituto "Nicolás Cabrera", Campus de Cantoblanco, 28049 Madrid, Spain
[7] Condensed Matter Physics Center (IFIMAC), Universidad Autónoma de Madrid, 28049 Madrid, Spain
[8] CNR-SPIN Uos L'Aquila, Via Vetoio 10, I-67100 L'Aquila, Italy
[9] CNR-IOM, TASC Laboratory, Area Science Park-Basovizza, 34139 Trieste, Italy
[10] Department of Physics, Indian Institute of Technology Kanpur, Kanpur, 208016, India
[11] College of Science, Institute of Materials Physics and Chemistry, Nanjing Forestry University, Nanjing 210037, P. R. China
[12] Theoretical Physics and Applied Mathematics Department, Ural Federal University, Mira Street 19, 620002 Ekaterinburg, Russia
[13] CNR-IMM Istituto per la Microelettronica e Microsistemi, VIII strada 5, I-95121 Catania, Italy
[14] INSTM, University of L'Aquila unit, 67100, L'Aquila (AQ), Italy

* Correspondence: antonio.politano@univaq.it;

S1. Theoretical Methods

Modelling of the atomic structure and energetics of gas adsorption on Pt_3Te_4 was carried out using the QUANTUM-ESPRESSO code [1] and the GGA−PBE functional with van der Waals (vdW) corrections, feasible for the studying of the adsorption of molecules on surfaces [2,3]. Energy cutoffs of 25 and 400 Ry for the plane-wave expansion of the wave functions and the charge density, respectively, and the 4 × 4 × 3 Monkhorst-Pack k-point grid for the Brillouin sampling were used [4]. For the modeling of the surface, we used slab of two Pt_3Te_4 layers each of these layers contain $PtTe_2$ and Pt_2Te_2 layers (see Figure 1). Note that in the slab of any number of Pt_3Te_4 layers on one surface will be Pt_2Te_2 layer and on opposite side $PtTe_2$. Moreover, we also considered the presence of the Te vacancies in top layer. To imitate contribution from rigid subsurface area of bulk crystals in the modeling of the surfaces, we performed optimization of the only atomic positions. In order to take into account the contribution from flexibility of nanosheets, we performed optimization of both atomic positions and lattice parameters. The enthalpies of physical adsorption were calculated by the standard formula:

$$\Delta H_{phys} = [E_{host+mol} - (E_{host} + E_{mol})]$$

Here, E_{host} is the total energy of the surface before adsorption, and E_{mol} is the energy of the single molecules of considered species in empty box. In the case of water adsorption, we only considered adsorption from the gaseous phase. Energy of chemical adsorption is defined as difference between the total energy of the system after and before decomposition of physically adsorbed molecule. For the case of physical adsorption, we also evaluated differential Gibbs free energy the formula

$$\Delta G = \Delta H - T\Delta S$$

where T is the temperature and ΔS is the change of entropy after formation molecule–substrate noncovalent bond, which was estimated similar to the gas → liquid transition and hence can be evaluated by the standard formula

$$\Delta S = \Delta H_{vap}/T$$

where ΔH_{vap} is the empirical enthalpy of vaporization.

All formulas and technical details for the calculations of the HER and OER are the same as used for the modeling of these reactions over a Pt(111) substrate [5,6].

References

1. Giannozzi, P.; Baroni, S.; Bonini, N.; Calandra, M.; Car, R.; Cavazzoni, C.; Ceresoli, D.; Chiarotti, G.L.; Cococcioni, M.; Dabo, I. QUANTUM ESPRESSO: a modular and open-source software project for quantum simulations of materials. *Journal of physics: Condensed matter* **2009**, *21*, 395502.
2. Perdew, J.P.; Burke, K.; Ernzerhof, M. Generalized Gradient Approximation Made Simple. *Phys. Rev. Lett.* **1996**, *77*, 3865-3868.
3. Barone, V.; Casarin, M.; Forrer, D.; Pavone, M.; Sambi, M.; Vittadini, A. Role and effective treatment of dispersive forces in materials: Polyethylene and graphite crystals as test cases. *Journal of Computational Chemistry* **2009**, *30*, 934-939, doi:10.1002/jcc.21112.
4. Monkhorst, H.J.; Pack, J.D. Special points for Brillouin-zone integrations. *Phys. Rev. B* **1976**, *13*, 5188-5192.
5. Greeley, J.; Jaramillo, T.F.; Bonde, J.; Chorkendorff, I.; Nørskov, J.K. Computational high-throughput screening of electrocatalytic materials for hydrogen evolution. *Nature materials* **2006**, *5*, 909-913.
6. Tang, Y.; Allen, B.L.; Kauffman, D.R.; Star, A. Electrocatalytic activity of nitrogen-doped carbon nanotube cups. *Journal of the American Chemical Society* **2009**, *131*, 13200-13201.
